# Treatment efficacy of *Thunbergia laurifolia*, *Curcuma longa*, *Garcinia mangostana*, and *Andrographis paniculata* extracts in *Staphylococcus aureus*-induced rabbit dermatitis model

**DOI:** 10.14202/vetworld.2022.188-197

**Published:** 2021-01-29

**Authors:** Charinya So-In, Nuchsupha Sunthamala

**Affiliations:** 1 Department of Veterinary Technology, Faculty of Agricultural Technology, Kalasin University, Kalasin 46000, Thailand; 2 Department of Biology, Faculty of Science, Mahasarakham University, Mahasarakham, 44150, Thailand.

**Keywords:** antibacterial, antidermatitis, anti-inflammatory, antioxidant, *Garcinia mangostana*, lipid peroxidation

## Abstract

**Background and Aim:**

Dermatitis is a soft-tissue infection caused by *Staphylococcus aureus*. The recurrence of inflammatory skin is linked to clinical manifestations. Anti-inflammatory cytokines, which are essential for tissue damage, are released by bacteria through skin tissues. Oxidative stress causes inflammatory cells to necrotize and reduces their antioxidant profile, resulting in toxic damage to surrounding tissues. Although studies on the antibacterial effects of *Thunbergia laurifolia* Lindl., *Curcuma longa* L., *Garcinia mangostana* L., and *Andrographis paniculata* (Burm.). Bacterial infection of *S. aureus* have been conducted, most of these studies have been *in vitro* and were not related to the rabbit model. In addition, anti-inflammatory and antioxidant studies need to be evaluated. Thus, this study aims to compare the antibacterial, anti-inflammatory, and antioxidant properties of four local herbs with a standard antibiotic in *S. aureus*-induced rabbit dermatitis model.

**Materials and Methods:**

The skin of New Zealand white rabbits were artificially wounded using a sterile blade and then infected with *S. aureus*. The rabbits were divided into seven groups, each with three rabbits (Total 21 rabbits): The first group was the no infection group (no infection and no treatment with scarification), the second group was the no treatment group (*S. aureus* infection of the wound but no treatment), and the other five treated groups were *T. laurifolia, C. longa, G. mangostana, A. paniculata*, and bacitracin cream, all of which involved wound infection and treatments. The treatment lasted for 7 days. The antibacterial, anti-inflammatory, and antioxidant properties after treatment were measured.

**Results:**

The efficacy of *T. laurifolia*, *C. longa*, *G. mangostana*, and *A. paniculata* was similar to that of an antioxidant and free radical scavenging property. The bacterial infection process gradually reduced the activities of antioxidant systems (i.e., enzymatic levels and gene expressions) and total glutathione. However, the activities of the antioxidant system were steadily increased when treated with herbal extracts. During bacterial invasion of the skin, the concentration of thiobarbituric acid reactive molecules, the level of lipid peroxidation, and the expression of anti-inflammatory cytokine genes were increased. All these were decreased when herbal extracts were used to treat the lesion.

**Conclusion:**

It can be concluded that *T. laurifolia, C. longa, G. mangostana*, and *A*. paniculate extract have antibacterial, anti-inflammatory, and antioxidant properties and are effective antibacterial agents. *G. mangostana* is the most effective herbal extract for antidermatitis and has the potential to be used as an alternative topical treatment.

## Introduction

The Gram-positive bacterium *Staphylococcus aureus* is a common causative agent for virulent dermatitis, mastitis, pododermatitis, epidemic abscesses, and septicemia in domestic rabbits [1-3]. In addition, *S. aureus* infection harms animal welfare and causes economic loss in industrial rabbits [4]. After infection, bacteria invade the host and then attach and remain on tissues, stimulating the immune system [5]. The host defends the infection by inducing the innate and adaptive immune responses on the host skin. The mechanism includes the induction of interleukin 1 (IL-1), tumor necrosis factor-α (TNF-α), antimicrobial peptides (β-defensins), pro-inflammatory cytokines, and chemokines [6,7]. In addition, the alteration of the mitochondrial immunology factor is also triggered. This process produces many reactive oxygen species (ROS), increasing oxidative stress, and lowering antioxidant ability [8,9]. Essential microorganisms in rabbits orchestrate food digestion and control the growth of the intestinal flora. Therefore, toxin production can kill the rabbit due to the loss of beneficial bacteria and possible overgrowth of pathogenic bacteria [10]. Penicillin and its derivatives have been widely used to treat *S. aureus* infections [11]. However, some studies have shown that *S. aureus* resists certain antibiotics, such as erythromycin, bacitracin, neomycin, lincomycin, and gentamicin [12,13].

Plant materials are used for anti-inflammatory, antimicrobial, and antioxidant purposes as a natural medical system because of their significant advantages of having fewer side effects and pathogenic tolerance than conventional antibiotics [14]. The phytochemical compounds found in the Thai herb *Thunbergia laurifolia* Lindl., belonging to the Acanthaceae family, include sterols (beta-sitosterol, stigmasterol, and alpha-spinasterol), phenolic compounds (gallic acid, apigenin, protocatechuic, and caffeic acid), carotenoid compounds (lutein), unclassified steroids, and glycoside [15]. In addition, *T. laurifolia* has a pharmacological effect on *S. aureus* infection and methicillin-resistant *S. aureus*, according to an *in vitro* study [16]. Curcumin, a polyphenolic compound with a yellowish color, is the most important phytochemical in *Curcuma longa* L. Several pharmacological activities have been investigated, including antimicrobial, anti-inflammatory, anticancer, and antioxidant properties. Curcumin inhibits bacterial cell proliferation by blocking the development of the Z-ring, which inhibits cytokinesis and mecA gene transcription, decreasing the expression of PBP2α proteins. In addition, the interaction of peptidoglycan and curcumin can damage *S. aureus* cell walls and membranes, resulting in cell lysis [17-19]. *Garcinia mangostana* L. (mangosteen), a Southeast Asian fruit, is well known for its antibacterial, antiseptic, anti-inflammatory, analgesic, antiparasitic, antipyretic, and anticarcinogenic properties [20]. In the mangosteen pericarp, compounds such as garcinone B, garcinone E, and a-mangostin are known as xanthones [21-23]. The major derivative of extractions showing active antimicrobial activity against the Gram-positive bacteria, *S. aureus*, is a-mangostin. Thus, the bacterial membrane is disrupted, resulting in intracellular material leakage [24]. In Asia, the extraction of *Andrographis paniculata* (Burm.) Wall., a member of the Acanthaceae family, is well known. The phytochemical compounds are diterpenes and lactones, with andrographolide being a primary extraction product with antibacterial, anti-inflammatory, and antioxidant properties [25,26].

Although studies on the antibacterial effects of *T. laurifolia*, *C. longa*, *G. mangostana*, and *A. paniculata* on bacterial infection of S. aureus have been conducted, most of them have been *in vitro* and did not use the rabbit model. In addition, anti-inflammatory and antioxidant studies need to be evaluated.

Thus, this study aims to compare the antibacterial, anti-inflammatory, and antioxidant properties of four local herbs with a standard antibiotic in *S. aureus*-induced rabbit dermatitis model.

## Materials and Methods

### Ethical approval

Eight-week-old New Zealand white rabbits were purchased from the National Laboratory Animal Center (Nakhon Pathom, Thailand) and housed according to the Animal Ethics Committee for Use and Care at Kalasin University, Kalasin, Thailand (approval number: KSU11/2559). Unless otherwise mentioned, the rabbits were housed with *ad libitum* food.

### Study period and location

This study was conducted from August 2016 to February 2017 at Kalasin University, Kalasin, Thailand.

### Experimental design and sample collection

The rabbits were divided into seven groups, each with three rabbits (for a total of 21 rabbits): The first group was the no infection group (no infection and no treatment with scarification), the second group was the no treatment group (*S. aureus* infection of the wound but no treatment), and the other five treated groups were *T. laurifolia*, *C. longa*, *G. mangostana*, *A. paniculata*, and bacitracin cream (Bacitracin Ointment USP, Phoenix Global Supply Group Inc., NY, U.S.A.), all of which involved wound infection and treatments.

For anesthesia, the rabbits were held in the anesthesia chamber with the following settings: 2-2.5% isoflurane (Isoflurane USP, Halocarbon Products Corporation, North Augusta, SC, U.S.A.) and2 L/min oxygen flow under standard atmospheric pressure. On the left flank, the infection site was carefully shaved using an electric trimmer. Scarification was performed on the prepared skin infection site. *S. aureus* (ATCC 6538) at 1×10^6^ colony-forming units (CFU)/mL was applied to the scarification wound for 48 h [27-29]. The herbals and bacitracin creams were applied to the treated groups 3 times a day at 07.00-08.00, 13.00-15.00, and 19.00-20.00. A daily photograph of the wound was taken to monitor the progress. After injection with pentobarbital sodium (Nembutal^®^, Ceva, Brussels, Belgium), the rabbits were euthanized 24 h later. The skin was rapidly excised and stored at −80°C for further examination.

### Preparation of T. laurifolia, C. longa, G. mangostana, and A. paniculata crude extracts

Air-dried and ground herbs (1000 g) were extracted with 80% ethanol at 25°C for 7 days. The crude extract was filtered and then evaporated under reduced pressure using a rotary evaporator R-II (Buchi Company, Flawil, Switzerland). The percentage yield was calculated by weighing the extracted herbs. High-performance liquid chromatography was used to perform a preliminary phytochemical analysis. The biologically active chemicals, including alkaloids, phenolics and tannins, flavonoids, anthraquinones, coumarin, saponins, terpenoids, steroids, and glycosides, were screened using previously described methods [30].

### Formulation of T. laurifolia, C. longa, G. mangostana, and A. paniculata cream

The crude extract was dried, and the solution was prepared with 50% ethanol to obtain the anticipated final dilutions of 5, 3.75, 3.125, 2.5, 1.875, and 1.25 mg/mL for antimicrobial (*S. aureus*) *in vitro* testing. These concentrations were processed using paper disks (5.5 mm diameter) compared with 50% ethanol and antimicrobial control disks containing amoxicillin, chloramphenicol, amikacin, and metronidazole. The four herbs were formulated as creams in a 10% concentration with a cream base following the extraction process. Its ingredients include chloramphenicol, Polysorbate 80 (5% w/v), stearyl alcohol (20% v/v), isopropyl myristate, liquid white paraffin (15% v/v), and propylparaben (0.02% w/w).

### Measurement of antibacterial activities

The homogenates were serially diluted 10-fold before being spread onto nutrient agar plates in 0.1 mL aliquots. The number of viable cells in the scarified wounds (CFU/g) was estimated after 48 h of incubation at 35°C.

### Determination of superoxide dismutase (SOD), catalase (CAT), and glutathione (GSH) peroxidase (GPx) activities

The level of SOD activity was determined using the inhibition degree of formazan formation [31]. The supernatant was extracted from an aliquot of homogenate mixed with chloroform and ethanol. The reagent mixture, including xanthine, 2-[2-[bis(carboxymethyl)amino]ethyl-(carboxymethyl)amino]acetic acid (EDTA), disodium carbonate (Na_2_CO_3_), 2-[2-methoxy-4-[3-methoxy-4-[3-(4-nitrophenyl)-5-phenyltetrazol-3-ium-2-yl]phenyl]phenyl]-3-(4-nitrophenyl)-5-phenyltetrazol-3-ium;dichloride, and bovine serum albumin, was mixed with the supernatant. After thoroughly mixing the mixture, xanthine oxidase was added. The same procedure was performed with a bovine CuZn-SOD standard for comparison purposes. Dichlorocopper was used to stop each reaction after being incubated for 20 min at 25°C. The formazan absorbance was measured at a wavelength of 550 nm. The degree of formazan inhibition was used to compare the supernatant with the SOD standard.

Before the reaction was stopped with ammonium molybdate, the homogenate was incubated in hydrogen peroxide (H_2_O_2_) substrate for 1 min at 37°C, and CAT activity was measured. The formation of a yellow complex was measured using absorbance at a wavelength of 405 nm and compared with the CAT standard.

The sample homogenate, 0.02 mM EDTA, 8.26 mM sodium azide, and 2.48 mM sodium phosphate buffer (pH 7.4) were incubated for 10 min at 30°C for GPx activity. After that, 1.24 mM reduced GSH was applied. H_2_O_2 (_1.24 mM) was used to initiate the reaction, and 3.31% (w/v) sulfosalicylic acid (SSA) was used to stop it. The reaction mixture was centrifuged at 1500 × g for 15 min. The supernatant was extracted to evaluate the GPx operation [31].

### Measurement of GSH oxidized, GSH reduced, and GSH content

The amount of GSH in the body was measured. The rabbit skin homogenates were deproteinized with SSA and centrifuged at 10,000 × g for 10 min at 4°C after being held for 10 min at 2-8°C. The supernatants were mixed with the reaction mixture (e.g., EDTA, potassium phosphate buffer (pH 7.0), GSH reductase, 5,5′-dithiobis-(2-nitrobenzoic acid) (DTNB), and nicotinamide adenine dinucleotide phosphate [NADPH]). The absorbance of the thiol anions was measured using a spectrophotometer at a wavelength of 405 nm every 60 s for 5 min.

The supernatant was mixed with the reaction mixture, which consisted of EDTA, NADPH, DTNB, and GSH reductase in phosphate-buffered saline (pH 7.0), to determine total GSH. The absorbance of the thiol anions was measured every 60 s for 5 min intervals at a wavelength of 405 nm using a UV (ultraviolet) microplate reader. The homogenate was treated with 4-VP before adding the reaction mixture, followed by incubation at 25°C for 1 h, to measure the oxidized GSH (GSH disulfide [GSSG]) content. Total GSH, GSH, and GSSG contents were determined by comparing the net slope of the sample with the slope of the standard curve of GSH or GSSG [32].

### Determination of lipid peroxidation

The thiobarbituric acid (TBA) assay was performed to determine lipid peroxidation. The reaction mixture (e.g., trichloroacetic acid, acetic acid, and 2-TBA) was added after the sample homogenate, and malondialdehyde (MDA) standard was incubated for 1 h at 37°C. Then, the samples were boiled for 15 min. The TBA reactive species (TBARS) were measured using a spectrofluorometer with an emission wavelength of 551 nm and an excitation wavelength of 528 nm [33].

### Real-time polymerase chain reaction (PCR) analysis of antioxidant gene and pro-inflammatory cytokine levels

The expression of rabbit mRNAs for antioxidant genes (CAT, GPx, CuZn-SOD, and Mn-SOD), pro-inflammatory cytokines (interferon [IFN]-γ, IL-10, TNF-α, and transforming growth factor [TGF]-β1), and the reference gene glyceraldehyde-3-phosphate dehydrogenase as a control was measured using real-time PCR. Total RNA was isolated from skin specimens using the RNA Easy kit (QIAamp Blood Kit; Qiagen GmbH, Hilden, Germany) following the manufacturer’s instructions. The RNA was dried and dissolved in 30 mL of nuclease-free distilled water before being stored at −80°C. RNA purity and concentration were monitored using a NanoDrop Spectrophotometer (Thermo Scientific, Waltham, MA, USA). The primer mixture, RNA template, and RNA preparation were all mixed.

In a 20 μL reaction with 12.5 μL SYBR Green and 0.25 M for each primer, real-time PCR was performed using 2 μL templates. The software requirements were preset based on the surveyed methods, with a few adjustments for real-time PCR (Applied Bio-system Thermo Fisher, Waltham, MA, USA). The quantitative mRNA expression level of targeted genes was estimated as follows: The cycle threshold (CT) – the number of PCR cycles needed for fluorescence to reach a high value that is far higher than background fluorescence. Relative gene expression differences were calculated using the 2^–ΔΔCT^ approach. Relative gene expressions were defined using the mean value±SD [34,35]. Table-1 shows the custom primer sequences.

**Table-1:** The specific primers used for real-time PCR.

Genes	Primers	Annealing temperature (°C/s)	Number of cycles	Product size (bp)
CuZn–SOD	5’–AAGGCCGTGTGCGTGCTGAA–3’	56/60	28	246
	5’–CAGGTCTCCAACATGCCTCT–3’			
Mn–SOD	5’–GCACATTAACGCGCAGATCA–3’	55/60	32	241
	5’–AGCCTCCAGCAACTCTCCTT–3’			
CAT	5’–GCAGATACCTGTGAACTGTC–3’	55/60	30	229
	5’–GTAGAATGTCCGCACCTGAG–3’			
GPx	5’–CCTCAAGTACGTCCGACCTG–3’	56/60	26	197
	5’–CAATGTCGTTGCGGCACACC–3’			
GAPDH	5’–TCCACTCACGGCAAATTCAACG–3’	64/30	22	145
	5’–TAGACTCCACGACATACTCAGC–3’			
TNF–α	5’–GACGTGGAAGTGGCAGAAGAG–3’	55/30	23	154
	5’–TGCCACAAGCAGGAATGAGA–3’			
IFN–γ	5’–CCAACGCAAAGCAATACATGA–3’	64/30	35	414
	5’–CCTTTTTCGCTTCCCTGTTTTA–3’			
IL–10	5’–GGTTGCCAAGCCTTATCGGA–3’	55/30	24	180
	5’–ACCTGCTCCACTGCCTTGCT–3’			
TGF–β1	5’–TGACGTCACTGGAGTTGTACGG–3’	55/30	25	201
	5’–GGTTCATGTCATGGATGGTGCTGF–3’			

IL=Interleukin, TNF=Tumor necrosis factor, SOD=Superoxide dismutase, GAPDH=Gene glyceraldehyde-3-phosphate dehydrogenase, PCR=Polymerase chain reaction

### Statistical analysis

The experimental findings were evaluated using a one-way analysis of variance with Tukey’s *post hoc* test (Statistical Package for the Social Sciences Inc., Chicago, IL, USA). p≤0.05 was considered statistically significant.

## Results

### Phytochemical screening of ethanol extracts

The phytochemical screening of *T. laurifolia*, *C. longa*, *G. mangostana*, and *A. paniculata* crude extracts showed the presence of alkaloids, phenolics, tannin, and terpenoids. *A. paniculata* crude extract was negative for the presence of flavonoids and coumarin. Only *G. mangostana* crude extract was positive for the presence of glycosides. All plant crude extracts showed the absence of anthraquinone, saponin, and steroids (Table-2).

**Table-2:** Phytochemical screening of ethanol crude extracts.

Phytochemical	*Thunbergia laurifolia*	*Curcuma longa*	*Garcinia mangostana*	*Andrographis paniculata*
Alkaloids	Positive	Positive	Positive	Positive
Phenolics and tannins	Positive	Positive	Positive	Positive
Flavonoids	Positive	Positive	Positive	Negative
Anthraquinones	Negative	Negative	Negative	Negative
Coumarin	Positive	Positive	Positive	Negative
Saponins	Negative	Negative	Negative	Negative
Terpenoids	Positive	Positive	Positive	Positive
Steroids	Negative	Negative	Negative	Negative
Glycosides	Negative	Negative	Positive	Negative

### Effect of crude extracts on the rabbit dermatitis lesions

Herbal-treated rabbits showed time-dependent wound changes in *S. aureus*-infected skin 6 days after infection. The cutting area of the skin was thickened, blunted, and edematous 1 day after exposure. The intensity of this reaction increased over 2 days. This was due to the fact that clinical improvements in all affected groups were identical when measured 2 days after S. aureus infection. The beneficial effects of *T. laurifolia*, *C. longa*, *G. mangostana*, *A. paniculata*, and bacitracin cream on dermatitis were evident (wound contraction) on days 5, 4, 3, 5, and 6, respectively.

**Figure-1:**
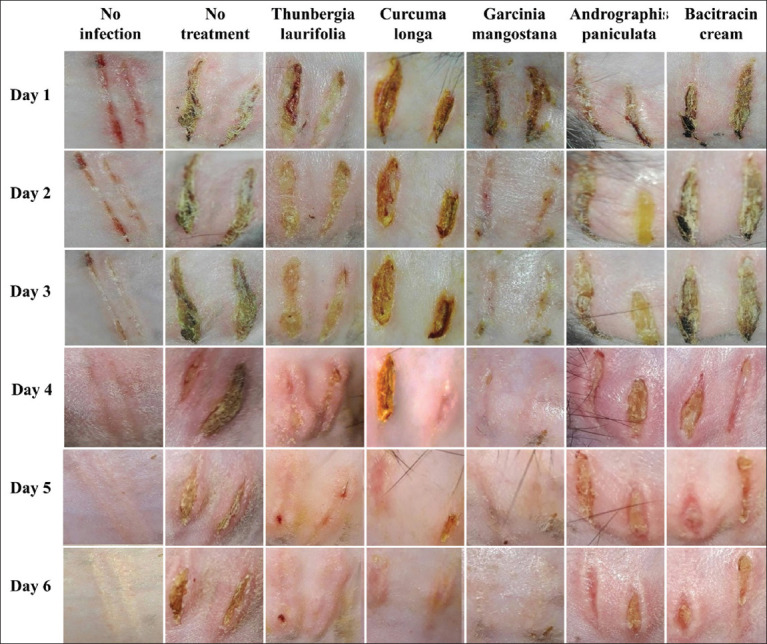
Effect of crude extracts on the rabbit dermatitis lesions. The lesion of rabbit skin in seven groups is as follows; no infection is uninfected and untreated with scarification group, no treatment is the bacterial infection wound. With the therapy, *the infected wounds are Thunbergia laurifolia*, *Curcuma longa*, *Garcinia mangostana*, *Andrographis paniculata*, and bacitracin cream.

Furthermore, there was no lesion in *G. mangostana*-treated group on day 4 after treatment. The wound was similar to that of the non-infected group (the most effective in inhibiting bacteria). The effectiveness of the other three herbs was comparable (Figure-1).

### Effect of crude extracts on antibacterial activities

In contrast to the control group, all herbs and bacitracin cream significantly decreased viable colony counts (Table-3). Surprisingly, the colony of *S. aureus* was significantly reduced in groups treated with *C. longa* and *G. mangostana*, which were similar to the antibacterial profile of bacitracin cream.

**Table-3:** Antibacterial activities of crude extracts and bacitracin cream in dermatitis rabbit skin.

Groups	Log CFU/g
No Infection	2.022±0.632^a^
No treatment	8.572±0.357^b^
*Thunbergia laurifolia*	5.810±1.032^c^
*Curcuma longa*	4.925±0.540^d^
*Garcinia mangostana*	4.819±0.440^d^
*Andrographis paniculata*	5.405±1.240^c^
Bacitracin cream	5.071±0.146^d^

CFU=Colony-forming unit.

### Effect of crude extracts on antioxidant systems

**Figure-2:**
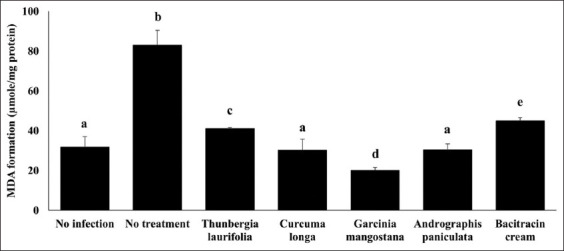
Effect of crude extracts on antioxidant genes expression. The antioxidant mRNA expression of dermatitis rabbit skins given herbals and bacitracin cream. The data are presented as the mean±SD (n=5). Different lowercase letters indicate a significant difference among different precipitation levels (p≤0.05).

**Figure-3:**
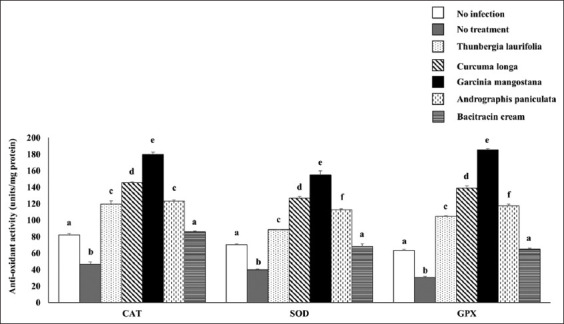
Effect of crude extracts on antioxidant enzyme activity. The antioxidant enzyme activity of dermatitis rabbit skins given herbals and bacitracin cream. The data are presented as the mean±SD (n=5). Different lowercase letters indicate a significant difference among different precipitation levels (p≤0.05).

**Figure-4:**
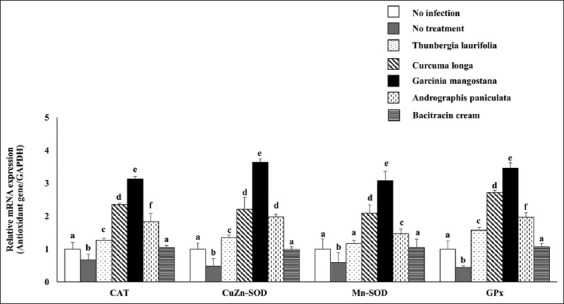
Effect of crude extracts on lipid peroxidation level. The lipid peroxidation level of dermatitis rabbit skins given herbals and bacitracin cream. The data are presented as the mean±SD (n=5). Different lowercase letters indicate a significant difference among different precipitation levels (p≤0.05).

The levels of CAT, GPx, CuZn-SOD, and Mn-SOD mRNA expression were significantly downregulated in the infection group as compared to the no infection group. However, the antioxidant genes were dramatically increased after using the four herbs and bacitracin cream (Figure-2). The enzyme activities of CAT, SOD, and GPx were significantly reduced in the no treatment group but significantly increased when the infected rabbits were treated with herbs and bacitracin cream, corresponding to the level of antioxidant genes (Figure-3). Throughout the infection, the lipid peroxidation level in the dermatitis rabbit skin was abnormally high due to the presence of MDA (Figure-4). All therapies substantially decreased MDA levels. Surprisingly, lipid peroxidation was significantly reduced in *G. mangostana*-treated group, in which it returned to normal levels. These findings support the efficacy of *G. mangostana* supplement cream’s antioxidant system in the dermatitis rabbit.

### Effect of crude extracts on total GSH and GSH/GSH disulfide ratio

The total GSH levels were lower in rabbits infected with S. aureus and induced dermatitis progression than in the control group. However, the total GSH levels of rabbits treated with four herbs were significantly increased, particularly *G. mangostana*, which was at the same level as the no infection group. Similarly, after *S. aureus* infection, the ratio of GSH and GSSG was significantly reduced, but it appeared to increase in *G. mangostana*-treated group (Table-4).

**Table-4:** The total GSH level and the ratio of GSH/GSSG of dermatitis rabbit skin treated crude extracts and bacitracin cream.

Groups	Total GSH	Reduced GSH	Oxidize GSH (GSSG)	Ratio GSH/GSSG
No infection	8.603±0.158^a^	6.045±0.113^a^	2.558±0.074^a^	3.145^a^
No treatment	3.618±0.157^b^	1.663±0.132^b^	1.355±0.056^b^	1.227^b^
*Thunbergia laurifolia*	6.760±0.272^c^	4.430±0.204^c^	2.330±0.098^a^	1.901^c^
*Curcuma longa*	7.903±0.028^a^	5.806±0.113^a^	2.097±0.047^a^	2.768^a^
*Garcinia mangostana*	10.524±0.293^d^	7.046±0.093^d^	2.278±0.078^a^	3.093^a^
*Andrographis paniculata*	7.382±0.294^c^	5.495±0.099^a^	1.887±0.069^a^	2.912^a^
Bacitracin cream	7.071±0.146^c^	5.547±0.066^a^	2.524±0.048^a^	2.197^c^

GSH=Glutathione, GSSG=Glutathione disulfide. The data are presented as the mean±SD (n=5). Different lowercase letters indicate a significant difference among different precipitation levels (p≤0.05).

### Effect of crude extracts on inflammatory profiles

**Figure-5:**
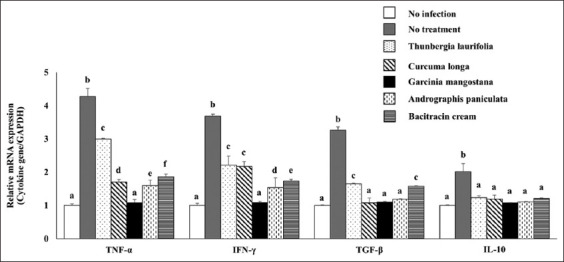
Effect of crude extracts on inflammatory cytokine mRNA expression. The inflammatory cytokine mRNA expression of dermatitis rabbit skins given herbals and bacitracin cream. The data are presented as the mean±SD (n=5). Different lowercase letters indicate a significant difference among different precipitation levels (p≤0.05).

Cytokine genes (e.g., TNF-α, IFN-γ, TGF-β, and IL-10 mRNA expressions) were significantly upregulated in *S. aureus* infection without treatment (Figure-5). In contrast to the control group, all four herbs substantially reduced the expression of the four genes. *G. mangostana* and *A. paniculata* significantly reduced the expression of inflammatory cytokines, TNF-α and IFN-γ. Only *G. mangostana*-treated group showed a noticeable reduction of all genes. In the dermatitis rabbit, these findings demonstrated the anti-inflammatory ability of *G. mangostana* supplementation.

## Discussion

Dermatitis is a recurrent inflammatory skin disease characterized by hyperepidermal proliferation, irregular differentiation, and inflammatory cell penetration into the dermis. Cellulitis is the clinical manifestation of superficial bacterial infection in this case. Several pro-inflammatory cytokines, such as TNF-α, IFN-g, TGF-b, and IL-10, were triggered in response to bacteria after infection. Alteration of immunological factors caused mitochondrial and skin cell dysfunction, resulting in ROS development, reduced antioxidant ability, and increased oxidative stress [9]. In different animal models, antioxidants provide one of the most important mechanisms for scavenging various ROS and inhibiting lipid peroxidation [36,37]. In bacterial infections, a rise in oxidants contributes to decrease antioxidant potential, resulting in imbalanced system and oxidative stress.

The study findings showed that the antioxidant condition (enzymatic and mRNA levels) was significantly reduced during the early stages of *S. aureus* infection, confirming previous findings regarding the relationship between skin lipid peroxidation and disease progression. Dermatitis is more vulnerable to oxidative stress as a result of this infection. GSH levels were lower during the dermatitis induction process. However, the function of cytokines in *S. aureus* infection-induced dermatitis has not been identified. This study showed mRNA expression of pro-inflammatory cytokines (IFN-γ, TNF-α, and TGF-β1) significantly upregulated in the infection, similar to IL-10, anti-inflammatory agents commonly used to reduce skin injury.

*T. laurifolia* pharmacological activity has been considered an antibacterial mechanism in *S. aureus* infection [8]. However, this study examined the herb’s antibacterial, antioxidant, and anti-inflammation activities in the skin of rabbits. In this study, the correlation between antioxidant activity and total phenolic content showed an increase in the antioxidant system and decreased lipid peroxide through ROS scavenging. These findings correspond to the studies by Phyu and Tangpong [38] and Jetawattana *et al*. [39], who reported that the total phenolic content (expressed as an effective antioxidant) was high in an aqueous leaf extract. In addition, increased expression of TGF-β, TNF-α, IFN-γ, and IL-10, a pro-inflammatory cytokine that responds to bacteria, was associated with an anti-inflammatory effect of this herb.

The antioxidant and anti-inflammatory properties of curcumin from *C. longa* are two key mechanisms that can explain the majority of curcumin’s effects on various diseases [18]. Curcumin is a highly effective lipid-soluble antioxidant that intercepts lipid radicals and converts them to phenoxyl radicals within the cell membrane. Curcumin has also been applied as a useful systemic indicator of oxidative stress [19]. Curcumin has been shown to increase antioxidant activities (e.g., SOD and GSH) and decrease lipid peroxides [17]. Therefore, curcumin’s effect can be defined as ROS scavenging. It is worth noting that all other studies back up our findings that curcumin may modulate the antioxidant mechanism in the neutralization of free radicals. It inhibits ROS by increasing total GSH and decreasing lipid peroxidation. Curcumin reveals a similar effect to *T. laurifolia*, which has an anti-inflammatory effect despite the high levels of pro-inflammatory cytokines.

α-Mangostin is another major extracted product of *G. mangostana*, which has been shown to protect against *S. aureus* infection by interrupting bacterial membranes, allowing the leakage of intracellular contents [24]. The key pharmacological effect of a-mangostin’s antibacterial activity is the suppression of inflammation and ROS oxidative enzymes activity [40,41]. The study findings revealed that α-mangostin improved the cellular antioxidant system and increased GSH in *G. mangostana*. The decline of TBARS, which indicates oxidative cellular damage on lipid peroxidation, suggests that the reduced ROS level indicates an increase in cellular protection. Our findings were also supported by Márquez-Valadez *et al*. [42], who found that α-mangostin significantly increased GPx activity in rat brain synaptosomes. In addition, Tsai *et al*. [41] discovered that a high-fat diet causes hepatic steatosis in rats by increasing cellular tension and that α-mangostin treatment reduces TBARS levels while increasing GPx, SOD, and GSH activities. In terms of anti-inflammatory effects, *G. mangostana* was identical to the other two herbs. However, *G. mangostana* had the highest pro-inflammatory cytokine expression.

In this study, andrographolide, which is the most common extract from *A. paniculata*, was found to have antioxidant and anti-inflammatory properties. There was a decline in pro-inflammatory cytokine and lipid peroxidation and increased antioxidant activities. In addition, Zhan *et al*. [43] found that andrographolide reduced MDA levels, increased CAT and SOD activity, and decreased the development of IL-1, IL-6, IL-10, and TNF-α in UV-irradiated mice.

## Conclusion

*T. laurifolia*, *C. longa*, *G. mangostana*, and *A. paniculata* crude extracts exhibited antibacterial, anti-inflammatory, and antioxidant activities. However, *G. mangostana* was the most effective and could be used as an alternative substance. This study reveals that the healing efficacy of *G. mangostana* crude extract was achieved by reducing the expression of antioxidant genes (CAT, SOD, and GPx) and inflammatory cytokine genes (TGF-β, TNF-α, IFN-γ, and IL-10) to levels similar to those of the no infection group. Thus, *G. mangostana* crude extract might be a potential topical *S. aureus* dermatitis agent. Further studies are needed to investigate the antimicrobial properties of *G. mangostana* crude extract against other bacterial pathogen-induced animal diseases.

## Authors’ Contributions

CS and NS: Conceived and designed the study, performed the study, analyzed the data, and drafted and revised the manuscript. All authors read and approved the final manuscript.
